# Morphological evolution of Ge/Si(001) quantum dot rings formed at the rim of wet-etched pits

**DOI:** 10.1186/1556-276X-7-601

**Published:** 2012-10-30

**Authors:** Martyna Grydlik, Moritz Brehm, Friedrich Schäffler

**Affiliations:** 1 Institut für Halbleiter-und Festkörperphysik, Johannes Kepler Universität, Altenbergerstr. 69, Linz, 4040, Austria

**Keywords:** SiGe, Quantum dots, Molecular beam epitaxy growth, Pit-patterned substrates

## Abstract

We demonstrate the formation of Ge quantum dots in ring-like arrangements around predefined {111}-faceted pits in the Si(001) substrate. We report on the complex morphological evolution of the single quantum dots contributing to the rings by means of atomic force microscopy and demonstrate that by careful adjustment of the epitaxial growth parameters, such rings containing densely squeezed islands can be grown with large spatial distances of up to 5 μm without additional nucleation of randomly distributed quantum dots between the rings.

## Background

Semiconductor nanodots and islands in the Ge/Si(001) system arranged in a closely spaced manner and located on well-defined positions have attracted interest over the last years [1-3]. While the ordering of the islands enables their addressability, the grouping of the islands introduces the possibility to create interacting quantum dots, i.e., quantum dot molecule (QDM) [3-7]. One of the well-described examples of QDMs are quantum fortresses - {105}-faceted structures - resulting from the growth of a Si-rich SiGe alloy on pit-patterned substrates [4-7].

Quantum dot molecules might provide a unique opportunity to study carrier interaction among quantum dots or be useful for quantum cellular automata applications [8,9].

Quantum dots grown on planar substrates have the disadvantage of island-island repulsion due to the compressive strain and the trenches introduced at the island’s periphery, which does not allow for an effective overlap of the heavy hole (HH) wave functions, located in the two dots, even if those are close. In fact, to create such an overlap, two dots would have to almost merge since, due to the Ge composition gradient [10], the HH states are located near the dots’ apex [11]. Such merging is very difficult to achieve on planar substrates. QDM may offer a possibility for transport experiments on coupled epitaxial quantum dots, extending the recent reports on transport through single SiGe quantum dots [12].

In a recent work, we have shown that islands nucleate at the rim of pits and mesas as soon as a critical angle (approximately 30°) between the pit/mesa-sidewall and the planar (001) surface is exceeded [13]. An additional requirement for the growth of quantum dot molecules and rings is that the growth temperature should not be too low. Otherwise, the surface migration of the Ge atoms is reduced, leading to island nucleation in the plateau regions in-between the pits. If such a plateau is not existing, as shown in a previous work, i.e., the {111} pits are touching each other, the Ge forms a wetting layer at the pit sidewalls with an inverted high aspect ratio dot in the middle of the pit [14].

In this paper, we describe the assembly of epitaxial semiconductor quantum dot arrays with position control, which is enabled by utilizing anisotropic wet etching as a mean of patterning the substrate.

In order to obtain densely squeezed islands arranged in a ring-like or zigzag manner, we utilize the preferential nucleation sites at the rim of steep, {111}-faceted pits in combination with high growth temperature and a Ge coverage that forms on a flat (001) surface which is a metastable wetting layer [15].

## Methods

High-resistivity Si(001) samples were covered with 70 nm of SiN_*x*_. Hereafter, fields with patterns of equidistantly spaced pits were defined by electron-beam lithography. The distances between the pits range from 1 to 5 μm, and the pre-patterned field size is 100 × 100 μm^2^.

Reactive ion etching was used to transfer the pattern into the SiN_*x*_ mask that hereafter plays the role of an etching mask for the anisotropic wet etchant tetramethylammonium hydroxide. Since the etching rates in the Si <001> directions are almost two orders of magnitude higher than the etching rate in <111> direction [16], pits with well-defined {111} facets are formed [14]. Since etching stops effectively as soon as the inverted {111} pyramid is achieved, the depth of the pit is defined by the opening’s size. On all fields the openings are square-shaped with a side length of 800 nm, except for one field where the side length is 400 nm. After removing the SiN_*x*_ hard mask and *ex situ* chemical cleaning (see also [17]), the sample was degassed *in situ* in the molecular beam epitaxy chamber at 700°C for 45 min. As a first growth step, a Si buffer layer of 45-nm thickness was grown at a substrate temperature ramped from 450°C up to 550°C with a growth rate of 0.6 Å/s. During a growth interruption, the substrate temperature was ramped-up to the Ge growth temperature of 700°C at which 3.8 ML of Ge was deposited (growth rate of 0.05 Å/s). We have chosen the value of 3.8 ML of Ge because for growth on flat Si (001) substrates at 700°C, this volume results in the formation of a metastable wetting layer thickness before the islands form (for experimental evidence and theoretical explanation see [15,17-19]). By using a pit pattern, we intentionally create material sinks towards which the Ge atoms in the topmost unstable monolayer are driven by surface diffusion (MG, unpublished work). To investigate the morphological evolution of the islands in more detail, we performed high-resolution atomic force microscopy (AFM) scans using tips with typical tip diameters of 2 nm and tip half-opening angles of 15°.

## Results and discussion

**Figure 1:**
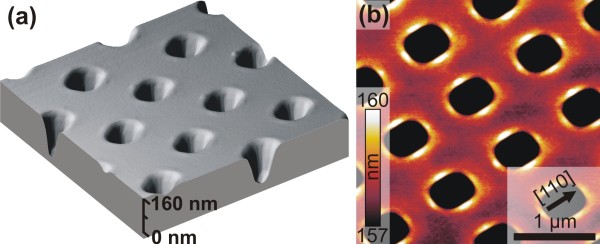
**Wetting layer thickening around steep pits.** (**a**) 3D AFM image of the pits after the growth of 3.8 ML of Ge and (**b**) a surface-sensitive AFM height image (scan size 3 × 3 μm^2^) of the Ge wetting layer growing on the pit-patterned field. Wetting layer thickening (bright color in (b)) can be observed in the <110> directions, i.e., where the pit has the steepest facets - {111}.

As the Ge-rich wetting layer (WL) (about 85% maximum Ge content at the Ge growth temperature used in this work [20]) is pseudomorphically strained on the flat (001) substrate, the pit geometry induces partial strain relaxation in the near vicinity of the rim of the pit, as discussed in detail in [13,21]. This extra relaxation leads to material accumulation, and, thus to an initial thickening of the WL. Figure 1a depicts a 3D AFM image of the pits after growth. The pits are slightly rounded at the intersections of the {111} planes (see also [14]), but the overall squared shape with dominant {111} facets remains. The thickening of the WL near the rim of the pit can be better seen in Figure 1b, where the same image is plotted in a surface-sensitive height mode. Evidently, the WL thickening is most efficient in the <110> direction (white regions around the pits) which is caused by the fact that the elastic relaxation of the WL is increased by the high curvature of the {111} pit-sidewall (pit-sidewall angle is 54.7° with respect to (001)). The AFM images depicted in Figure 1a,b were taken at the middle of the field with a pattern period of 1 μm. The growth conditions for the WL and the islands are not independent of the position on the field. The volume stored in the islands and the WL is decreasing as we move away from the interface between the patterned field and the surrounding flat surface ((MG, unpublished work), [22]). A full description of the Ge surface diffusion processes happening at the periphery of the pre-patterned field is described elsewhere (MG, unpublished work). Since the pits act as material sinks, as can be seen for instance by the thicker WL, material diffuses from the planar area towards and into the patterned field. The length scale at which this diffusion happens can be up to tens of micrometers (MG, unpublished work), [22] depending on the growth temperature and more importantly, on the growth rate. Thus after growth the planar areas surrounding patterned field less than 3.8 ML are effectively stored and no islands are observed, whereas in the outer pit rows of the patterned field, considerably more than 3.8 ML are accumulated and consequently, islands are formed there.

**Figure 2:**
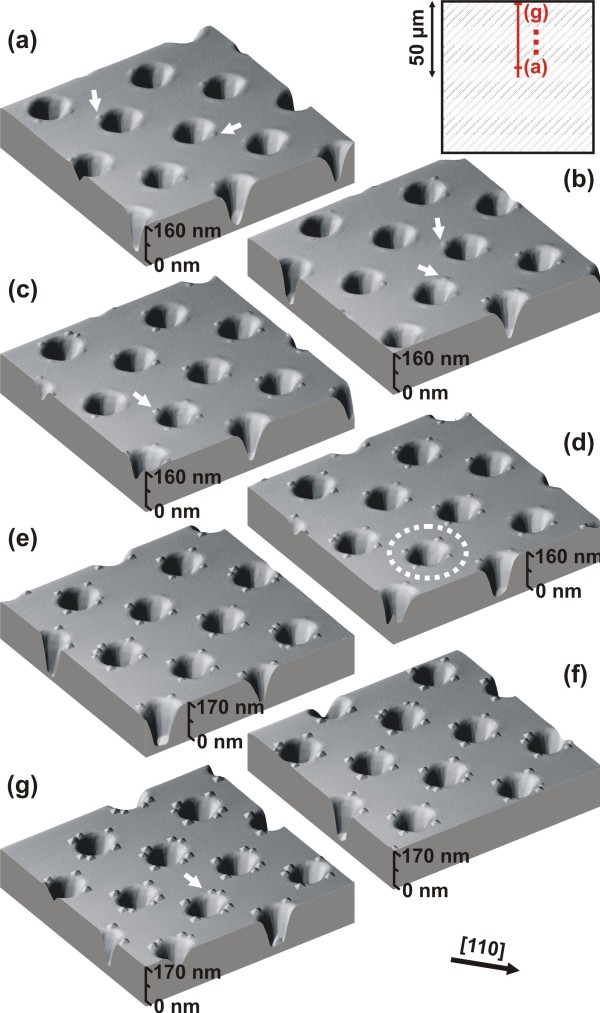
**Position-dependent AFM images.** Three-dimensional AFM images, recorded on the patterned field on the positions as indicated in the inset on the upper right corner of the figure starting from close to the middle of the field (**a**) and approaching the border (**b,c,d,e,f,g**). The white arrows highlight positions of the islands in the <110> directions at the rim of the {111} pits. Progressive nucleation of the islands at the at the four pit-edges is highlighted by the white dashed ring at one example pit.

Figures 1a 2 show 3D AFM scans taken at different positions, starting in the exact middle of the patterned field (Figure 1a) and approaching the border of the field (Figure 2a,b,c,d,e,f,g). One can see that small islands, indicated by the white arrows in Figure 2a,b,c, progressively continue to nucleate at the four edges in the <110> directions (see the dotted circle in Figure 2d) where the initially formed WL was relaxed the most and where it had the highest thickness.

Closer to the pattern field edge, there is enough Ge available to form fully faceted pyramidal islands that, once all four-edge positions in the <110> directions are occupied, continue to nucleate on other pit-rim positions (see arrow in Figure 2g).

Vastola et al. [13] have shown that island nucleation at the rim of a pit with steep sidewall inclinations (>30°) is favorable due to energetic reasons. The island can relieve the strain energy into the substrate underneath most efficiently, if it is located at the rim of the pit. In addition, for steep pits the WL is more relaxed due to an increased surface curvature [13,21]. Atoms diffusing on the substrate surface must pass through this pit-rim region prior to ending in the pit. The low local chemical potential is increasing the probability of clustering and nucleation of 3D islands.

**Figure 3:**
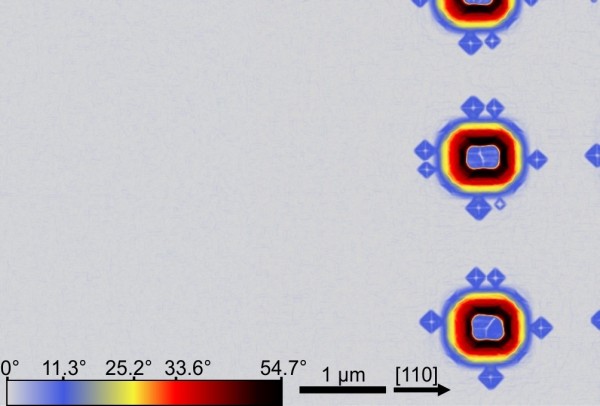
**Border of the patterned field.** AFM surface angle image, recorded on the border between patterned field and planar surrounding. No island formation is observed on the planar part of the sample. The deposited Ge volume (3.8 ML) is below the critical thickness for island nucleation on planar substrates (4.2 ML).

**Figure 4:**
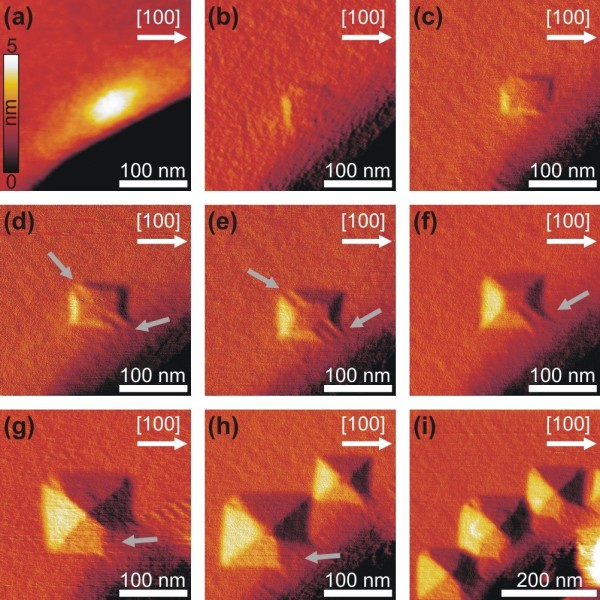
**High resolution AFM images of the islands’ morphology evolution.** 280 × 280 μm^2^ and 400 × 400 μm^2^ AFM images in derivative mode. Evolution of the islands from the thickening of the wetting layer at the rim of the pit in [110] direction (**a**) to pre-pyramids (**b,c,d**), truncated pyramids (**e,f**), full pyramids (**g,h,i**) and transition domes (i). In order to better use the strain, relaxed WL in the very close vicinity of the pit rim pyramid islands split its symmetry during formation. The additional facets to the {105} ones in panels (g,h,i) are most probably {116} facets (gray arrows). The evolution towards those {116} facets happens in a complex manner through high-index facets. Those might be {9 13 77}, {3 4 26}, and {5 7 40}, but their true shape is almost impossible to determine by means of AFM since their respective facet areas are very small. Note the different length scale in panel (i).

In Figure 2 one can see small islands (mounds) nucleating at the rim of the pit in the <110> directions. As we move towards the edge of the field, those mounds evolve into larger islands, while the planar surrounding of the pattern field remains fully island-free, as can be seen in Figure 3 where the local surface slope with respect to the (001) surface is plotted. The resulting islands’ morphological evolution is shown in Figure 4, where the scan sizes of the AFM micrographs in height mode (Figure 4a) and derivative mode (Figure 4b,c,d,e,f,g,h,i) are 280 × 280 nm^2^. In Figure 4i the scan size was chosen to be 400 × 400 nm^2^ in order to visualize the successive decoration of islands around the pit rim at higher coverage. Figure 4a shows the thickening of the wetting layer at the <110> directional edges of the pit. This material accumulation is completely unfaceted and, therefore, we have chosen a surface-sensitive height mode representation since else the image contrast would be too low.

In Figure 4b,c the first but not fully faceted islands, called ‘pre-pyramids’ [23], are presented. Even though those islands only consist of a small fraction of the typical {105} facets, their overall squared base shape is a precursor of the later evolution into pyramids.

An interesting observation was made for those developing pyramids. With an ongoing growth evolution, the intersections of the {105} planes (which is in [110] direction - at the pit rim and opposite to it) split into a ‘tweezers’-like structure (see gray arrows in Figure 4d,e). The tweezers-like splitting of the truncated pyramid side edge that is opposite to the pit becomes less pronounced as the {105} facets of the pyramids become fully developed (see Figure 4f,g). Finally, the tweezers-like structure close to the {111} facet of the pit evolves into a {116} facet (inclination of 13.26° with respect to (001)), as determined by nano-goniometric analysis using the methods shown in [24]. Those {116} facets build about 9% of the total sidewall surfaces of the islands. It is not easy to determine all the facets involved in the tweezers-like structures, since their facet area is very small. Using the nano-goniometric analysis as given in [24], we conclude that the facets are of relatively high index like {3 4 26} and {5 7 40}.

Close to the corner of the field (as seen in Figure 4i) the islands successively decorate the whole rim of the pit and some of the islands even evolve into so-called ‘transition domes’ [25] that have also steeper facets than the {105} ones of the pyramid.

**Figure 5:**
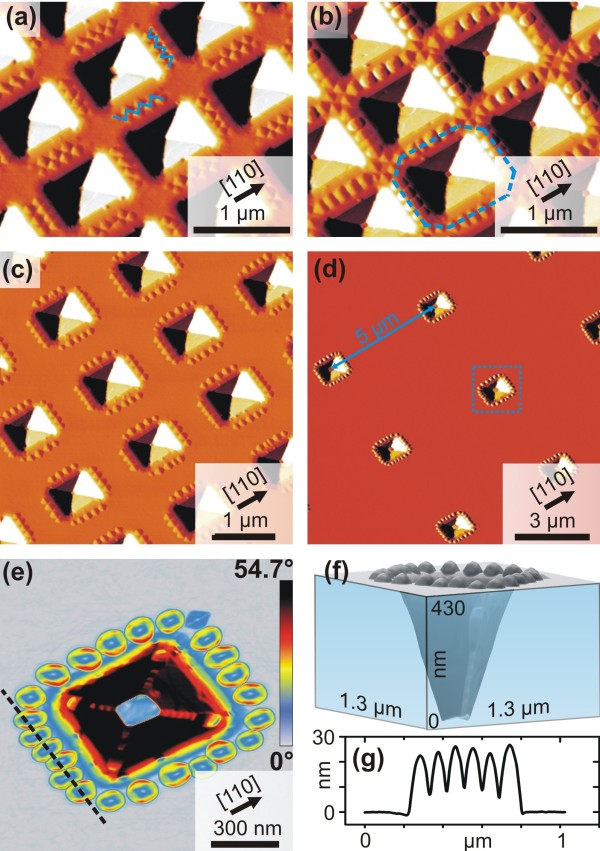
**Rim-bound islands for higher pit periods.** (**a,b,c,d**) AFM images in derivative mode of islands grown around the pits taken from fields with pit spacing of 1 μm (a,b), 1.5 μm (c), and 5 μm (d). Panels (**e,f**) show one representative pit decorated with islands that is indicated by the dotted square in (d) in a surface angle image and in a 3D image.

Figure 5a,b presents two spots on the same field (1 μm pit period, but larger pit opening length as compared to the previously mentioned pits) where the amount of accumulated material was altered utilizing the aforementioned border effect [22], while keeping the pit size and the spacing of the pits constant. For lower coverage (Figure 5a), the islands align in a zigzag structure in between the pits, while for higher coverage, i.e., closer to the border of the field, the extra Ge transforms the islands into bigger transition domes and domes that align in a straight manner. In both cases the islands are densely packed.

In order to address a single cluster of islands around a pit, it becomes necessary to control their spacing. In Figure 5c,d we show that for a pit spacing of 1.5 μm and, more impressively, for 5 μm pit spacing, the islands still form densely packed arrays around the pit, while no isolated islands are nucleated between the pits. Note that for all samples in this work, 3.8 ML of Ge was deposited at 700°C. To achieve such islands organization, it is not only important to carefully adjust the amount of deposited material (metastable wetting layer thickness [15,17-19]), but also the growth rate should be low enough (0.05 Å/s in this case) so that material can diffuse to the material sinks at the rims of the pits. If the thickness of the WL between the pits is kept below its critical value and the growth rate is low, there is no upper limit of the inter-pit spacing for the ring-like island structures. Figure 5e,f shows an AFM scan of the single pit highlighted in Figure 5d by a dashed square. In Figure 5e the local surface slope with respect to the (001) surface is plotted, and the scale bar was chosen in such a way that the {105}, {113}, {15 3 23}, and {111} facets appear in blue, yellow, red, and black, respectively. Thus, one sees that the vast majority of the islands around the rim is of merged, dome-like structure.

Figure 5g shows the line scan across the path given by the dashed line in Figure 5e. The domes are so close together that they actually merge at their base. This is interesting because dome islands grown on planar substrates usually exhibit a certain distance to each other, even if the density is high [17]. This effect can be called island-island repulsion. The reason for this behavior is that the islands strain the substrate compressively at their periphery, which makes further accumulation of Ge at such lattice sites unfavorable [26]. On the investigated samples, shallow mounds nucleate close to each other around the rim. Due to the mounds’ relatively small volume, a large part of their base is located on the partly relaxed WL. Thus their position is fixed before they transform into larger pyramids and finally into merging domes. This is obviously a stronger effect than the island-island repulsion.

## Conclusions

We proposed a method for controlling the spatial ordering of SiGe quantum dots, which utilizes nucleation at preferential sites at the rim of the steep {111}-faceted pits. Such circularly ordered quantum dots might be useful for improved photoluminescence properties since it is possible to create areas with high local dot density [27].

For the applications as described in [12], it is necessary to obtain only two merging islands. Confining and merging islands around a pit seems to be a promising route to overcome the island-island repulsion and to create a system of two or more islands that can communicate with each other. For this purpose, the sidewalls of the pits should be steep (>30°) after wetting layer growth, which might be a shortcoming for contacting. But this problem might be solved by fabricating very small, but still steep pits as can be done for example by ultraviolet nanoimprint lithography with anisotropically remastered molds [28] or by focused ion beam patterning [2].

## Competing interests

The authors declare that they have no competing interests.

## Authors’ contributions

MG and MB contributed equally to the work in designing the growth of the samples, carrying out the AFM experiments and writing the manuscript. FS participated in the manuscript design and coordination. All authors read and approved the final manuscript.

## Authors’ information

MG and MB are post-docs and FS is a professor at the Institut für Halbleiter- und Festkörperphysik, Johannes Kepler Universität, Austria.
